# Comparison of Efficacy of Suprapatellar and Infrapatellar Approaches for Intramedullary Interlocking Nailing of Tibia in Patients With Tibial Fracture

**DOI:** 10.7759/cureus.74682

**Published:** 2024-11-28

**Authors:** Siva Swaminathan Santhanam, Sabaresh Velayutham, Ponnilavan Krishnan, Antony Albert, Brinda Ramanujam

**Affiliations:** 1 Orthopedics, Ysbyty Gwynedd, Bangor, GBR; 2 Orthopedics, Pondicherry Institute of Medical Sciences, Pondicherry, IND; 3 Information Technology, Mandayam Osuri Parthasarathi Vaishnav College for Women, Chennai, IND

**Keywords:** fracture fixation, infrapatellar approach, intramedullary nailing, intraoperative outcomes, suprapatellar approach, surgical technique, tibial fracture

## Abstract

Introduction: Intramedullary interlocking nailing is a common surgical procedure for tibial fractures, enabling early patient mobilization. Traditionally, the infrapatellar approach has been used for intramedullary interlocking nailing of tibial fractures, but the suprapatellar approach is gaining attention for its potential benefits. This randomized controlled study aimed to compare the duration of the surgery, intra-operative blood loss, and fluoroscopy time between the suprapatellar and infrapatellar approaches.

Methods: We included 38 adult patients with tibial shaft fractures, excluding those with non-union, open fractures, revision surgery, or low Glasgow Coma Scale (GCS). Patients were divided equally into two groups using block randomization: Group A (19 patients) underwent the infrapatellar approach, and Group B (19 patients) underwent the suprapatellar approach. Blood loss was measured using the gravimetric method and by changes in pre-operative and post-operative hemoglobin levels. Surgical duration by calculating the time elapsed between the start and end of the procedure and fluoroscopy time was logged from the fluoroscopy machine.

Results: In group A, blood loss averaged 154±30.98 mL, slightly more than in group B (150±32.92 mL), though the difference was not statistically significant (p>0.05). Group A also showed a higher difference in hemoglobin levels (2.20±1.13 g/dL) compared to group B (1.15±0.93 g/dL), which was statistically significant (p=0.02). Fluoroscopy time and duration of the surgery were slightly longer in group A compared to group B but not statistically significant (p=0.693).

Conclusion: The suprapatellar approach results in less blood loss, potentially promoting faster recovery, reduced need for blood transfusions, and shorter hospital stays. It also entails shorter fluoroscopy time and surgical duration, though not statistically significant, which may reduce radiation exposure for the surgical team.

## Introduction

The tibia is responsible for bearing more than 80% of body weight [1]. The incidence of tibial shaft fractures is 16.9 per 100,000 per year, with a higher rate in males at 21.5 per 100,000 per year, compared to 12.3 per 100,000 per year in females. Most tibial fractures are treated surgically as they allow early patient mobilization. Conservative treatment is reserved for the treatment of closed, stable, isolated, minimally displaced fractures caused by low-energy trauma [2,3]. Intramedullary interlocking nailing is considered a standard surgical treatment for closed fractures of the tibial diaphysis. The advantages of intramedullary nailing are reduced hospital stay, early full weight bearing, and early fracture healing. Also, it spares the extra-osseous blood supply, acts as a load-sharing device, and more importantly avoids extensive soft tissue dissection [4]. Infrapatellar approach has traditionally been used for the treatment of tibial fractures. However, in proximal tibial fractures, inserting a nail using the infrapatellar approach is challenging as the knee in flexion or hyperflexion causes the proximal fracture fragment to extend due to the quadriceps mechanism, whereas the distal fragment remains flexed, resulting in a procurvatum deformity of the tibia. The suprapatellar approach, introduced by Tornetta and Collins [5], helps reduce varus and procurvatum deformities in proximal tibia fracture because the semi-extended position may eliminate the force of the quadriceps and pes anserinus.

Many studies showed superior results in the suprapatellar approach with respect to post-operative outcomes such as functional knee outcome, and malalignment as compared to the infrapatellar approach [6-8]. Whether the suprapatellar approach provides a superior outcome in terms of intraoperative parameters like total blood loss, fluoroscopy time, and duration of the surgery compared with the infrapatellar approach remains controversial. This study was conducted to compare total blood loss, fluoroscopy time, and duration of the surgery between the suprapatellar approach and infrapatellar approach for the treatment of tibia shaft fracture.

## Materials and methods

Participants and randomization

This was a randomized prospective study conducted at our medical college after the study protocol and amendments were approved by the Pondicherry Institute of Medical Sciences (PIMS) institutional ethics committee (IEC: RC/2021/45). The study took place from December 2021 to December 2022, over a period of one year. A total of 38 patients, aged over 18 years, with tibial fractures presenting to the Orthopedics outpatient department and casualty, were selected and divided into Group A and Group B using a computer-generated block randomization technique. Patients were excluded if they had a low Glasgow Coma Scale (GCS), required revision surgery, had open fractures, or had tibial non-union. Patients in Group A underwent infrapatellar nailing of the tibia, while those in Group B underwent suprapatellar nailing of the tibia, after obtaining valid written informed consent.

Surgical procedure and data collection

For all patients, baseline data (age, gender, diabetic and hypertensive history) and injury characteristics (mechanism, fracture location, fracture pattern) were noted. All surgeries were performed by senior orthopedic surgeons, who were well-trained in both the infrapatellar and suprapatellar nailing techniques. Both groups were operated without the application of a tourniquet. All tibial fractures were fixed using interlocking intramedullary nails (Medtronic, Minneapolis, MN, USA) after fracture reduction and reaming of the intramedullary canal. Intraoperative parameters like blood loss, fluoroscopy time, and duration of the surgery were noted.

Intraoperative blood loss was measured using the gravimetric method. The dry weight of surgical sponges was recorded in grams before the start of the operation, and the wet weight was noted at the end of the procedure. Blood loss in the sponges was calculated as the difference between the wet and dry sponge weights, with each gram of weight difference corresponding to 1 milliliter of blood loss. Blood loss by suction was determined by subtracting the volume of irrigation fluid (in milliliters) from the total volume collected in the suction canister. Hemoglobin levels were compared pre-operatively and post-operatively to assess the hemoglobin difference: hemoglobin difference = pre-operative Hb - post-operative Hb. The total fluoroscopy time during the surgery was automatically displayed on the C-arm machine and recorded for each patient.

Statistical analysis

All data were summarized as mean ± standard deviations for continuous variables and as frequency (percentages) for categorical variables. Student’s t-test was used to compare the difference between those two groups for normally distributed data. The difference between those two groups was assessed with the Mann-Whitney U test for non-normally distributed data. p<0.05 was regarded as a significant difference.

## Results

In the present study, the amount of blood loss was observed to be higher in Group A (154±30.98 mL) compared to Group B (150±32.92 mL); however, the difference between the groups was not statistically significant (p-value > 0.05). The reduction in hemoglobin levels post-operatively was more pronounced in Group A (2.20±1.13 g/dL) compared to Group B (1.15±0.93 g/dL), with the difference in pre-operative and post-operative hemoglobin levels between the groups found to be statistically significant (p-value = 0.02). Furthermore, the fluoroscopy time was slightly higher in Group A (1.55±0.87 minutes) compared to Group B (1.52±0.62 minutes), though this difference was not statistically significant (p-value = 0.693). Similarly, the duration of the surgery was marginally longer in Group A (1.71±0.44 hours) compared to Group B (1.69±0.62 hours), but this difference was also not statistically significant (p-value = 0.693) (Tables 1-3).

**Table 1:** Demographic data of patients IP - infrapatellar approach, SP - suprapatellar approach

		Group A (IP)	Group B (SP)
Gender	Male	14 (73.7%)	18 (94.7%)
	Female	5 (26.3%)	1 (5.3%)
Fracture type	Proximal third	2 (10.5%)	2 (10.5%)
	Middle third	9 (47.4%)	7 (36.8%)
	Distal third	8 (42.1%)	10 (52.7%)
AO/OTA classification	42A	16 (84.2%)	14 (73.7%)
	42B	2 (10.5%)	4 (21%)
	42C	1 (5.3%)	1 (5.3%)
Diabetes		4 (21%)	2 (10.5%)
Hypertension		2 (10.5%)	3 (15.7%)
Blood thinners		-	-

**Table 2:** Distribution of patients based on age IP - infrapatellar approach, SP - suprapatellar approach

	Group A (IP)	Group B (SP)	P-value
Age	41.31±18.56	39.53±14.33	0.942

**Table 3:** Intraoperative parameters IP - infrapatellar approach, SP - suprapatellar approach

Parameters	Groups	Mean	Standard deviation	Z value	P-value
Blood loss (mL)	Group A (IP)	154	30.9838	-0 .672	0.502
	Group B (SP)	150	32.916		
Hb difference (g/dL)	Group A (IP)	2.2	1.13284	-3.071	0.002
	Group B (SP)	1.1474	0.93355		
Fluoroscopy time (mins)	Group A (IP)	1.5532	0.87426	-0.394	0.693
	Group B (SP)	1.5237	0.62332		
Duration of the surgery (hours)	Group A (IP)	1.7053	1.7053	-0.395	0.693
	Group B (SP)	1.6932	1.6932		

## Discussion

Most tibial fractures in our study were caused by road traffic accidents, with a clear male predominance. Of the 38 participants, 28 were male (73.6%) and 10 were female (26.4%). This aligns with the findings of Larsen et al. [2], where males had a higher incidence of tibial fractures (21.5/100,000/year) compared to females (12.3/100,000/year). In our study, the mean age of the population was 40.24±16.56 years, with males having a mean age of 37.96±16.61 years and females 47.3±14.24 years. This also mirrors the study by Larsen et al. [2], which noted that males tend to experience tibial fractures at a younger age, with the highest frequency between ages 10 and 20 years, while in females, the highest frequency occurs between ages 30 and 40 years.

In our study, the average intraoperative blood loss in the infrapatellar approach was 154±30.98 mL, while in the suprapatellar approach, it was 150±32.92 mL, indicating that the suprapatellar approach had slightly less intraoperative blood loss compared to the infrapatellar approach, though this difference was statistically insignificant. A study by Lu et al. [9] showed similar results, with blood loss in the suprapatellar approach (56.3±14.6 mL) being less than in the infrapatellar approach (60.5±9.3 mL). However, a contrasting result was found in a study by Sun et al. [10], where blood loss in the suprapatellar approach (22.11±7.13 mL) was higher compared to the infrapatellar approach (21.67±7.07 mL). In both studies, however, the differences in blood loss were statistically insignificant.

The hemoglobin difference refers to the variation between the hemoglobin level at the time of admission and the hemoglobin level on postoperative day 1. It is an indirect way of measuring blood loss. In our study, the average hemoglobin difference in the infrapatellar approach was 2.20±1.13 g/dL, while in the suprapatellar approach, it was 1.15±0.93 g/dL, indicating a smaller difference compared to the infrapatellar approach. This difference was statistically significant (p-value = 0.02), suggesting that the suprapatellar approach resulted in reduced blood loss compared to the infrapatellar approach. This is a more reliable and indirect method for estimating blood loss than the gravimetric method, as the gravimetric method only calculates blood loss in the sponges and suction. It does not account for blood loss on the sterile drapes, scrub dresses, instruments, and the floor.

In our study, the average fluoroscopy time in the infrapatellar approach was 1.55±0.87 minutes, while in the suprapatellar approach, it was 1.52±0.62 minutes, indicating that fluoroscopy time was slightly lesser in the suprapatellar approach compared to the infrapatellar approach. However, the difference between the groups was statistically insignificant (p-value = 0.693). A study by Al-Azzawi et al. [11] showed similar results, though they used radiation dose (dose area product) as a parameter. The radiation dose was lower in the suprapatellar approach (36±28.6 cGy·cm²) compared to the infrapatellar approach (76.33±33.4 cGy·cm²) and it was statistically significant (p<0.05). A study by Sun et al. [10] also demonstrated similar findings, with fluoroscopy time in the suprapatellar approach (80.61±37.24 seconds) being significantly shorter than in the infrapatellar approach (118.68±40.23 seconds) and it was statistically significant (p<0.05). The reduced fluoroscopy exposure time in the suprapatellar group may be attributed to the table setup, where the knee is positioned in a semi-extended state, allowing for a more accurate nail entry point and better fracture reduction. This results in fewer radiographs being needed to check nail positioning and fracture reduction. The suprapatellar approach also minimizes the time required to switch between anteroposterior and lateral radiographs, as it eliminates the need for limb repositioning.

In our study, the infrapatellar approach had a longer duration of the surgery compared to the suprapatellar approach. The duration of the surgery in the infrapatellar approach was 1.71±0.44 hours, while in the suprapatellar approach, it was 1.69±0.62 hours. Although the infrapatellar approach had a slightly longer surgical time, this difference was statistically insignificant (p-value = 0.693). Studies by Lu et al. and Al-Azzawi et al. [9,11] reported similar findings, where the duration of the surgery was shorter in the suprapatellar approach than in the infrapatellar approach, and both studies found these differences to be statistically significant (p-value <0.05). A study by Sun et al. [10] also showed similar results, with the duration of the surgery in the suprapatellar approach being 71.01±5.98 minutes, shorter than the infrapatellar approach (73.26±4.03 minutes), though this difference was statistically insignificant (p-value = 0.183).

This study has several limitations. First, the small sample size may restrict the generalizability of the findings to a larger population. Additionally, the involvement of multiple surgeons in performing the surgeries could introduce variability and potential bias. Lastly, the lack of patient follow-up limits the ability to assess long-term outcomes.

## Conclusions

In conclusion, the suprapatellar approach may be superior to the infrapatellar approach for intramedullary nailing of the tibia due to reduced blood loss, shorter fluoroscopy time, and shorter surgical duration. However, further studies are necessary to confirm these findings.
